# Inward rectifier potassium (Kir) channels mediate salivary gland function and blood feeding in the lone star tick, *Amblyomma americanum*

**DOI:** 10.1371/journal.pntd.0007153

**Published:** 2019-02-07

**Authors:** Zhilin Li, Kevin R. Macaluso, Lane D. Foil, Daniel R. Swale

**Affiliations:** 1 Louisiana State University Agricultural Center, Department of Entomology, Baton Rouge, LA, United States of America; 2 Louisiana State University, School of Veterinary Medicine, Department of Pathobiological Sciences, Baton Rouge, LA, United States of America; Hebrew University Hadassah Medical School, ISRAEL

## Abstract

**Background:**

Tick feeding causes extreme morbidity and mortality to humans through transmission of pathogens and causes severe economic losses to the agricultural industry by reducing livestock yield. Salivary gland secretions are essential for tick feeding and thus, reducing or preventing saliva secretions into the vertebrate host is likely to reduce feeding and hinder pathogen life cycles. Unfortunately, the membrane physiology of tick salivary glands is underexplored and this gap in knowledge limits the development of novel therapeutics for inducing cessation of tick feeding.

**Methodology:**

We studied the influence of inward rectifier potassium (Kir) channel subtypes to the functional capacity of the isolated tick salivary gland through the use of a modified Ramsay assay. The secreted saliva was subsequently used for quantification of the elemental composition of the secreted saliva after the glands were exposed to K^+^ channel modulators as a measure of osmoregulatory capacity. Lastly, changes to blood feeding behavior and mortality were measured with the use of a membrane feeding system.

**Principal findings:**

In this study, we characterized the fundamental role of Kir channel subtypes in tick salivary gland function and provide evidence that pharmacological inhibition of these ion channels reduces the secretory activity of the *Amblyomma americanum* salivary gland. The reduced secretory capacity of the salivary gland was directly correlated with a dramatic reduction of blood ingestion during feeding. Further, exposure to small-molecule modulators of Kir channel subtypes induced mortality to ticks that is likely resultant from an altered osmoregulatory capacity.

**Conclusions:**

Our data contribute to understanding of tick salivary gland function and could guide future campaigns aiming to develop chemical or reverse vaccinology technologies to reduce the worldwide burden of tick feeding and tick-vectored pathogens.

## Author summary

Tick feeding results in negative health and economic consequences worldwide and there has been continued interest in the development of products with novel mechanisms of action for control of tick populations. Kir channels have been shown to be a significant ion conductance pathway in arthropods and are critical for proper functioning of multiple biological processes. Previous work on insect Kir channels has focused on their physiological roles in renal system of mosquitoes and the data suggest that these channels represent a viable pathway to induce renal failure that leads to mortality. Based on the functional and cellular similarities of arthropod salivary glands and Malpighian tubules, we hypothesized that Kir channels constitute a critical conductance pathway within arthropod salivary glands and inhibition of this pathway will preclude feeding. Data presented in this study show that pharmacological modulators of Kir channels elicited a significant reduction in the fluid and ion secretory activity of tick salivary glands that resulted in reduced feeding, altered osmoregulation, and lead to mortality. These data could guide the future development of novel acaricides, RNAi, or genetically modified ticks to mitigate health and economic damages resulting from their feeding. Further, these data indicate a conserved function of Kir channels within multiple tissues of taxonomically diverse organisms, such as ticks and humans.

## Introduction

The neuroendocrinology and genetic regulation of tick salivary glands has been researched extensively in an effort to identify novel acaricide target sites that can alleviate the burden of tick-borne pathogens [1–10]. Unfortunately, the significant advancements in knowledge relative to tick genomics, tick saliva proteins, and vaccine technologies have translated poorly into successful control efforts. Compounding tick control efforts is the increase in tick-borne bacterial infections. For example, rickettsial diseases are steadily increasing within the Americas [11] and further, recent studies have shown that the most predominant human biting tick, *Amblyomma americanum*, is capable of acquiring, maintaining, and transmitting *Rickettsia rickettsii* [12]. The steady increase in tick populations and pathogens [13], increased vector competency of human biting ticks for rickettsial diseases[12], and the movement toward an epidemic of tick-transmitted pathogens [14,15] highlights the significance of research aimed to identify novel mechanisms of control to curb the health and economic burden of ticks.

Arthropod feeding results from the harmonious function of multiple organ systems that include olfactory and gustatory signaling to detect the food source, pharyngeal and cibarial pumps to generate a sucking action to imbibe fluid, and the salivary glands to secrete bioactive proteins that serve a variety of functions [16,17]. The documented importance of salivary secretions has stimulated attempts to develop vaccines against proteins in the secreted saliva to control tick [18] and horn fly [19] infestations of cattle and most recently, to prevent successful feeding of mosquitoes [20]. Interestingly, the efforts of previous vaccine development programs have primarily focused on the proteins secreted into the saliva and few efforts [21] have focused on the mechanisms enabling saliva secretion from the salivary gland.

The tick salivary gland is multifunctional and performs a key role in two events during blood feeding. First, the tick salivary gland secretes many bioactive proteins that are critical for acquisition of the blood meal [22] and cementing the tick onto the host, suggesting that inhibition of salivary gland function will reduce blood feeding efficacy. Second, the salivary gland is responsible for maintaining a proper salt and water balance during blood feeding. Mammalian blood contains high concentrations of sodium and potassium salts that would be toxic to the tick without coordinated osmoregulatory mechanisms during feeding, which is performed through the salivary gland by returning about 65–70% of the fluid and ion content of the blood meal back into the host [23]. Importantly, failure to osmoregulate would alter the concentration of ions in the hemoceal and result in inhibition of cellular functions, functionally making the blood meal toxic in a similar manner described after inhibition of mosquito Malpighian tubules [24,25]. Further, arthropod saliva is the media through which pathogens are horizontally transferred to the vertebrate host. We hypothesize that inducing salivary gland failure will 1) prevent feeding, 2) prevent osmoregulation during blood feeding to induce acute mortality, and 3) prevent pathogen transmission through reduced salivary secretions. However, prior to developing products, it is necessary to understand the mechanisms of ion transport across the membranes of the salivary gland acini and how modulation of these pathways influences saliva and feeding.

Potassium ion channels are a fundamental component of cellular physiology since they are responsible for establishing and maintaining the membrane potential of animal cells and serve crucial roles in cellular regulation [26]. In mammalian salivary glands, saliva secretion is associated with a pronounced efflux of K^+^ ions from the acinar cells, which results from an increase in basolateral membrane permeability to K^+^ ions [27,28]. In particular, inward rectifier potassium (Kir) channels and ATP-sensitive Kir (K_ATP_) channels are critical for saliva secretion and regulation of the ion concentrations by maintaining the cellular membrane potentials of the mammalian salivary gland acini [29–31]. Although Kir and K_ATP_ channels are well characterized in multiple mammalian tissues [32,33] and are exploited as drug targets [34], an understanding of the physiological role, expression patterns, and toxicological potential of these ion channels in ticks, and other arthropod disease vectors, is significantly less developed. Yet, the existing base of work suggests these channels serve significant roles in various arthropod tissues [35–40] and represents a putative insecticide target site [25], illustrating the need for continued characterization of these channels.

Kir channels function as biological diodes due to the unique biophysical property that allows the flow of potassium ions in the inward direction more easily than the outward direction at hyperpolarizing potentials [41], which allows the cell to return to the resting potential by increasing the intracellular concentration of cations. Kir subunits lack the S4 voltage sensor region that is responsible for gating in all voltage-dependent ion channels; thus, Kir channels are constitutively active if regulator mechanisms, such as sulphonylurea receptors or ATP, are absent. All Kir channels share similar molecular architecture and are tetramers assembled around an aqueous membrane-spanning pore that are gated by polyvalent cations that occlude the pore at cell potentials more positive than the K^+^ equilibrium potential [42,43]. K_ATP_ channels are a sub-type of Kir channel superfamily and are octomeric complexes of four pore-forming Kir channel subunits and four regulatory sulfonylurea receptor (SUR) subunits [32]. Contrary to “constitutively active” Kir channels, K_ATP_ channels are regulated by the ratio of intracellular ATP:MgADP, and thus couple the membrane potential to the metabolic state of the cell [44]. In pancreatic beta-cells, when the ATP:MgADP ratio is elevated, K_ATP_ channels close to prevent K^+^ efflux that results in membrane depolarization and Ca^2+^-entry through voltage sensitive Ca^2+^-channels. Conversely, K_ATP_ channels open when the nucleotide ratio is decreased, which results in K^+^ ion efflux that leads to membrane hyperpolarization and cessation of Ca^2+^-entry.

A gap in knowledge regarding the role of K^+^ ion channels in invertebrate salivary gland function and thus, saliva generation and secretion, has limited the ability to develop products that prevent tick blood feeding. To address this gap in knowledge, our group has aimed to characterize the role of Kir channels in the feeding cascade and salivary gland function of a medically relevant tick, *A*. *americanum*. Our previous work [35] and others [45] clearly illustrates the importance of Kir channels for proper salivary gland function and feeding of arthropods, which highlights the potential for these ion channels to be targeted by vaccine or chemical technologies in arthropods of medical significance. Therefore, the overarching goal of this investigation was to leverage multidisciplinary approaches to test the hypothesis that salivary gland function and feeding of *A*. *americanum* is reliant upon Kir channels expressed in the salivary gland. Knowledge gained from this study may be used to broadly guide future development of novel synthetic insecticides, RNAi, or transgenic organisms to mitigate human health concerns and curb economic losses that result from tick feeding.

## Methods

### Pharmacological modulators and reagents

The Kir channel inhibitor VU041, VU937 (inactive analog of VU041), VU625, and VU688 were originally discovered in a high-throughput screen (HTS) against the *Anopheles gambiae* and *Aedes aegypti* Kir1 channels, respectively [25,46]. These molecules were ordered by custom synthesis from Molport (Rita, Latvia, Europe). VU590 and VU608 were initially discovered in a HTS campaign targeting human Kir1.1 channels [47] and were purchased from Tocris Bioscience or synthesized by Vanderbilt Center for Neuroscience Drug Discovery, respectively. All K_ATP_ channel modulators were purchased from Sigma-Aldrich (St. Lewis, MO, USA). The inactive analog to VU0071063, termed VU063-I, was generously provided by Dr. Jerod Denton (Department of Anesthesiology, Vanderbilt University) and was subsequently purchased by custom synthesis from Molport (Rita, Latvia, Europe). All chemicals were designated to be >98% pure.

Hanks Balanced Salt Solution (HBSS) with calcium chloride and magnesium chloride was purchased from Life Technologies (14025–092) and was used in all Fluid secretion (Ramsay) assays (Ramsay, 1954). Dimethyl sulfoxide (DMSO) was purchased from Sigma-Aldrich (St. Louis, MO, USA). Clear 100% silicon II Chaulk (General Electric, M90050) was purchased from Ace Hardware and was used for the construction of the feeding membrane.

### Arthropod Rearing, Dissections, and Fluid collection

*A*. *americanum* adults were purchased from the Oklahoma State University Tick Rearing Facility (Department of Entomology; Stillwater, OK, USA) and were declared by the supplier to be free of all known pathogens. Adult ticks were approximately 1 month old at the time of analysis. Prior to allowing the ticks to blood feed, the ticks were stored for 14 days in an incubator at 28° C and 60% RH. Female salivary glands of partially blood fed ticks were dissected under a stereomicroscope in Hanks Buffered Salt Solution (HBSS) and the secreted ion and saliva was collected using a modified Ramsay assay [48]. Saliva secretion was stimulated by bath application of 100 μM dopamine HCl. Methods followed those that were previously published [7]. Concentration-response curves (CRC) were established with one gland of the tick exposed to dopamine only (control) and the second gland treated with the small-molecule modulator prior to dopamine expose, which enabled a paired statistical analysis.

### Arthropod feeding assays

**Fig 1:**
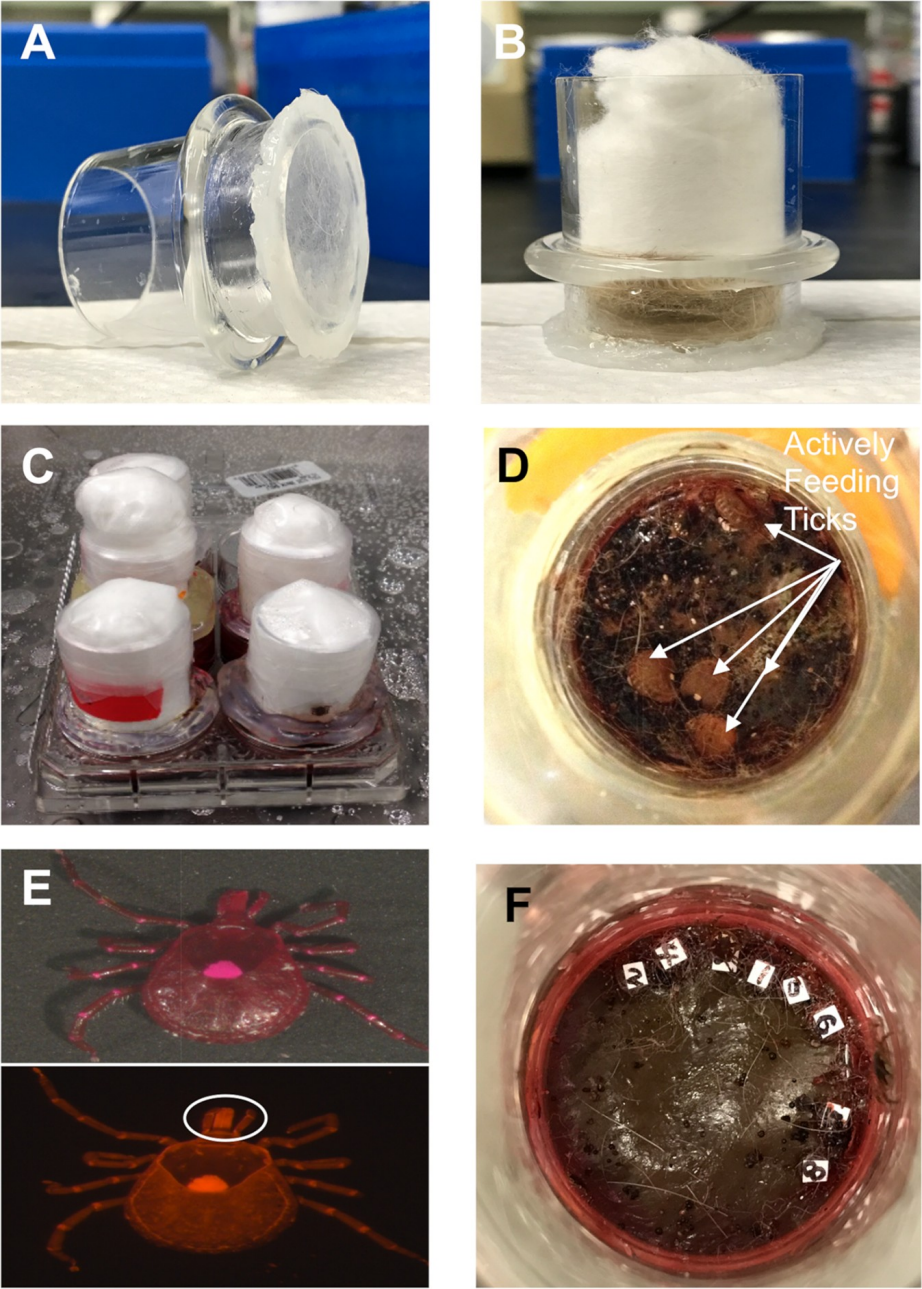
Artificial host feeding system used for *A*. *americanum* blood feeding. Individual components and fully constructed membrane feeding chambers are shown in panels **A-C** and actively feeding ticks are shown in panel **D**. Fluorescent images were captured of ticks to ensure the mouthparts (white circle) were fluorescent, indicating blood feeding due to inclusion of Rhodamine B into the blood meal (**E)**. Individual ticks were labeled with a numerical value and various parameters were assessed every 12 hours (**F**).

The physiology and gene expression profile is different between an unfed, feeding, and fed ticks [49,50], thus all studies were performed on partially fed ticks to normalize gene expression amongst individuals. To obtain partially fed ticks, we adopted an artificial host system that enables blood feeding of multiple ticks on a silicone membrane in lieu of an animal host, which has been previously described [51] and is shown in Fig 1A–1D. Briefly, the membrane consisted of 10 g silicone glue (Silicone II) was mixed evenly with 3g silicone oil AR 20 (Sigma-Aldrich; St. Louis, MO, USA) and 2g Hexane (Sigma-Aldrich; St. Louis, MO, USA). The silicone mixture was applied onto the lens paper purchased from Tiffen Company LLC (Hauppauge, NY, USA). The mixture was spread evenly using a glass spreader to an approximate thickness of 60–80 μm [51]. The membrane was placed at room temperature for 14–21 days prior to use. The constructed feeding chamber is shown in Fig 1A and 1B.

Glass chambers (28 mm outer diameter, 2 mm wall thickness, 45 mm cylinder with a 35 mm OD bead that is 12 mm up from the bottom of cylinder) were ordered from Greatglass (Wilmington, DE, USA) and were used to construct the tick-feeding chamber. A membrane that was dried for >14 days were glued on the chamber with the same silicone glue at least one day before loading ticks. Glued chambers were placed into six-well cell culture plate with water to ensure the membrane did not leak. A total of 10 females and 5 males were loaded into one chamber with freshly shaved heifer hair. Cotton balls were inserted into the top of the chamber and approximately 1 cm of head space was maintained for tick feeding (Fig 1C). After testing for leaks, the artificial membrane was immersed in 5 ml defibrinated bovine blood that was poured into a 6-well cell culture plate and tick feeding was allowed to commence immediately upon placement into the chamber (Fig 1D). The blood was purchased from Hemostat Laboratories (Dixon, CA, USA). Gentamycin and ATP were added to the blood at a concentration of 5 μg/ml and 1μM, respectively. The fluophore, rhodamine B (100 ppm), was included into the blood to ensure that all ticks included in data analysis had punctured the membrane and attempted to feed (Fig 1E). Ticks without fluorescent mouthparts (Fig 1E, white circle) were not included in the analysis. Ticks were monitored every 12 hours and individual ticks were tracked over time by numerically numbering each tick (Fig 1F). Feeding chambers were maintained in blood bath at 38°C with a 16 h:8 h light:dark photoperiod. The blood was changed twice a day at 12 h interval for the duration of the experiment.

### Tick fluid secretion assays

**Fig 2:**
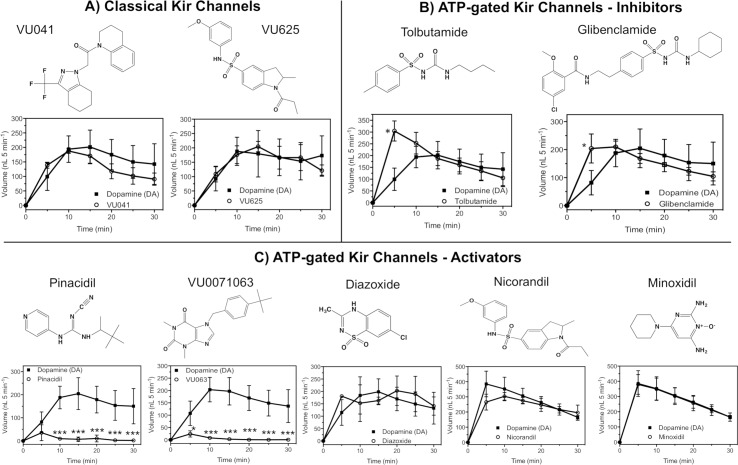
Influence of Kir channel modulators to the secretory activity of the *A*. *americanu*m isolated salivary gland. The influence of classical Kir channel modulators (A), K_ATP_ channel inhibitors (B), and K_ATP_ channel activators (C) were studied to determine the influence on saliva secretion. Each data point represents a mean (n: 3–5) volume of saliva secreted after 30-minute exposure to modulator followed by stimulation with 100 μM dopamine. Each treatment group was paired to a dopamine control from the same tick. * represents statistical significance at P<0.05 as determined by a paired t-test for each time point.

For measurement of secreted fluid, we employed the Ramsay assay that was initially developed to characterize the physiology of insect Malpighian tubules and the role of various membrane bound proteins to urine formation, urine secretion, and osmoregulation [48]. Modifications to enable measurements of secreted saliva of ticks have been previously described and used in this study [6,7,9]. Partially engorged female ticks (weighing 10–20 mg, 3 days blood-fed on membrane) were prepared from the artificial feeding system described above [51]. Salivary glands and the corresponding ductwork were dissected from the partially fed tick and then incubated in HBSS buffer for 1 h before initiating the Ramsay assay. After incubation, a single female gland was immersed in 15 μl HBSS buffer containing a HBSS + DMSO (vehicle control) or compound solubilized in HBSS. The main duct was drawn across a narrow grease dam made of high vacuum grease (Dow Corning Corporation, Midland, MI, USA) that was approximately 1 mm in height and immobilized on the surface of a petri dish. After drawing the main duct across the grease dam, the entire gland was submerged in HBSS and saliva secretion was stimulated by application of 100 μM dopamine HCl dissolved in HBSS. Basal levels of dopamine mediated saliva secretion measurements were taken in the first 5 minutes after preparation of the fluid secretion assay. A micro-injector (Nanoliter 2010, World Precision Instrument, Inc., Sarasota, FL, USA) controlled by a micro-syringe pump controller (Micro4, World Precision Instrument, Inc., Sarasota, FL, USA) was used for withdrawing the secretion formed at the tip of the duct in the heavy mineral oil, and the volume withdrawn was recorded in every 5 min for 30 min. To study the influence of K^+^ channel modulators (Fig 2) on saliva secretion, the gland was incubated in the compound solution for 30 minutes prior to exposure to dopamine that initiates salivation. Concentration-response curves were performed with one gland of the tick exposed to dopamine only (control) and the second gland treated with the Kir channel modulator prior to dopamine exposure, which increased the rigor of the experimental design through paired analysis. Total salivation for each time point of the treated glands was compared to the volume secreted at the same time point for dopamine only treated glands to obtain percent saliva secreted when compared to control. For the ATP challenge experiment, glands were incubated in HBSS buffer containing both 500 μM ATP and 300 μM Pinacidil/1 μM VU063 for 30 min before dopamine exposure.

### Analysis of tick saliva secretion data

The salivation time course data presented for the CRCs were collected using paired salivary glands where one salivary gland was treated with dopamine only and the other salivary gland was treated with the small-molecule modulator plus dopamine. The ability to pair the glands negated the variability due to factors that influenced individual ticks, such as blood meal size and age. The data points for each time point represent an average where n = 3–5 and the means for each time point of the treated glands was statistically compared to the same time point of the control (dopamine only treatment) by a paired t-test. Statistical significance was assessed based on P<0.05.

The concentration required to inhibit secretion by 50% (IC_50_) values were determined through the generation of concentration-response curves that were constructed with 5–6 concentrations. The percent salivation for each concentration of pinacidil/VU063 was determined by the formula: (secreted volume of gland treated with chemical + dopamine / secreted volume of gland treated with dopamine only) * 100. Each comparison was made from paired glands and the data points for each concentration represent the average % salivation of 3–5 paired glands. IC_50_ values were calculated by nonlinear regression (variable slope) using a Hill equation in GraphPad Prism (GraphPad Software, San Diego, CA, USA).

For the ATP challenge experiment, bars represent mean (n = 3–5) volume of secreted saliva over a 5 min period from 15–20 minutes while the error bars represent SEM. A one-way ANOVA with a multiple comparisons post-test was performed to determine statistical significance compared to dopamine only treatment.

### Quantification of tick blood feeding and mortality assessments

Ticks fed on control blood (vehicle only) or treatment blood (pinacidil or VU0071063) were monitored every 12 hours and the time of attachment, the time of detachment, and the time of reattachment was recorded for each tick. The changes in feeding biology was determined by quantifying the number of detachments per tick and the time until the first detachment for the treated groups compared to the non-treated control group. The average detachment per tick per feeding event was determined by counting the number of detachments for each female tick in the feeding chamber until mortality or completion of feeding. Mortality was not considered a detachment. The total number of detachments per tick was summed in each chamber and the detachment rate for 3 feeding chambers were averaged (n: 30–40 individuals). To quantify the volume of imbibed blood, individual unfed ticks were labeled as described before and were weighed to the nearest 0.1 mg. Ticks were subsequently removed from the feeding chamber after 1-, 2-, 3-, 4-, 5-, and 6-days of known feeding. Ticks that detached or died prior to the required feeding time were discarded from data analysis. At the predetermined time point, the attached tick was removed from the membrane and immediately weighed to determine the change in mass where an increase of 1 mg in total weight corresponded to 1 μL of blood ingested. Ingestion was corrected for the specific gravity (1.030 at 37°C) of bovine blood. Tick mortality was defined as a tick completely non-responsive to mechanical stimuli and only ticks that were previously attached were included in this measurement.

### Elemental analysis of tick saliva

The secreted saliva was analyzed via scanning electron microscopy/energy-dispersive X-ray spectroscopy (SEM-EDS) with a silicon drift detector (SDD) to obtain qualitative elemental composition and concentration of dopamine only-, pinacidil-, and VU0071063- treated glands. A Dual-Beam Focused Ion Beam (FIB) SEM equipped with EDS (EDAX) at the LSU Socolofsky Shared Instruments Facility (SIF) was used to image and analyze each dried saliva spot. The parameters of the image was set to be 10 kV and 5.7 nA current. We used EDAX TEAM software to acquire spectra to identify the Na^+^, K^+^, and Cl^-^ ions based on the characteristic X-ray. Due to the use of a silicon background, silicon was excluded from the analysis and only Na^+^, K^+^, and Cl^-^ were used for concentration calculations, but the value of silicon was used as an internal control. The concentration of each element was determined by the construction of a standard curve that was developed by spotting NaCl (in mM: 20, 50, 100, 200, 400, 600, 800) and KCl (in mM: 2, 4, 8, 16, 32, 64, 100) onto the silicon substrate with differing concentrations. The standard curves for each ion were generated based on atomic percentage values of each concentration, which were compared to the standard curves to enable calculation of the relative concentration of the elements. The atomic percentages of nine saliva droplets (1 nL) were determined using the SEM-EDS equipment and averaged to obtain a mean percent atomic for each individual time point and treatment group. The mean was compared to the percent atomic of the standard curve that was generated for each element (Na^+^, Cl^-^, and K^+^) and the concentration of total Na^+^, Cl^-^, and K^+^ in the whole saliva droplet was determined with the formula for a linear regression (y = mx + b). The molar concentration of the element was multiplied by the dilution factor that stemmed from the reconstitution of the dried saliva and then divided the product by the total volume of saliva secreted. This resulted in the total concentration of each element per nL of secreted saliva.

## Results

### Influence of K_ATP_ channel modulators to the secretory activity of the isolated *Amblyomma americanum* salivary gland

Here, we used the moderately developed pharmacological library of insect Kir channels [24,25,46,52] to test the influence of Kir channels to the secretory activity of the tick salivary gland. The volume of secreted saliva after exposure to VU041 and VU625 was not significantly (P>0.05) different at any time point when compared to dopamine only control glands (Fig 2A). The pharmacological profile of human K_ATP_ channels is well characterized [53] and enabled a more complete examination of tick K_ATP_ channels with multiple structural scaffolds of inhibitors and activators. The K_ATP_ inhibitors tolbutamide and glibenclamide were found to have minimal impact on the volume of secreted saliva at the 10–30 minute time points, but significantly (P<0.01) increased secretory activity at 5-minutes by approximately 3-fold when compared to paired control glands (Fig 2B).

Select pharmacological activators of K_ATP_ channels were shown to nearly abolish the secretory activity of the isolated salivary gland at a discriminatory concentrations ranging from 500 μM to 1 mM (Fig 2C). Pinacidil was shown to prevent nearly all secretory activity at time points ranging from 10 min to 30 minutes with the 5- minute time point producing the largest volume of secreted saliva at an average of 30 ± 12 nL, which was significantly (P<0.05) reduced when compared to dopamine-only treated (control) glands (81 ±11 nL/5 min) at the same time point (Fig 2C). The second K_ATP_ agonist studied, VU0071063, was shown to have a near identical pattern of inhibition when compared to pinacidil, but at a 3-fold less concentration. VU0071063 treated glands secreted less than 3 nL of saliva at the 10–30 minute time points and only secreted an average (n = 3) of 23 ± 9 nL of saliva whereas the paired control glands secreted 107 ± 21 nL at the 5-min time point, a statistically significant (P<0.001) reduction (Fig 2C). Diazoxide, nicorandil, and minoxidil are three structurally diverse activators that have varying specificity for human K_ATP_ channel subtypes, but were found to not influence the secretory activity of the isolated tick salivary gland (Fig 2C).

### Validation that reduced secretory activity is through K_ATP_ channel modulation

**Fig 3:**
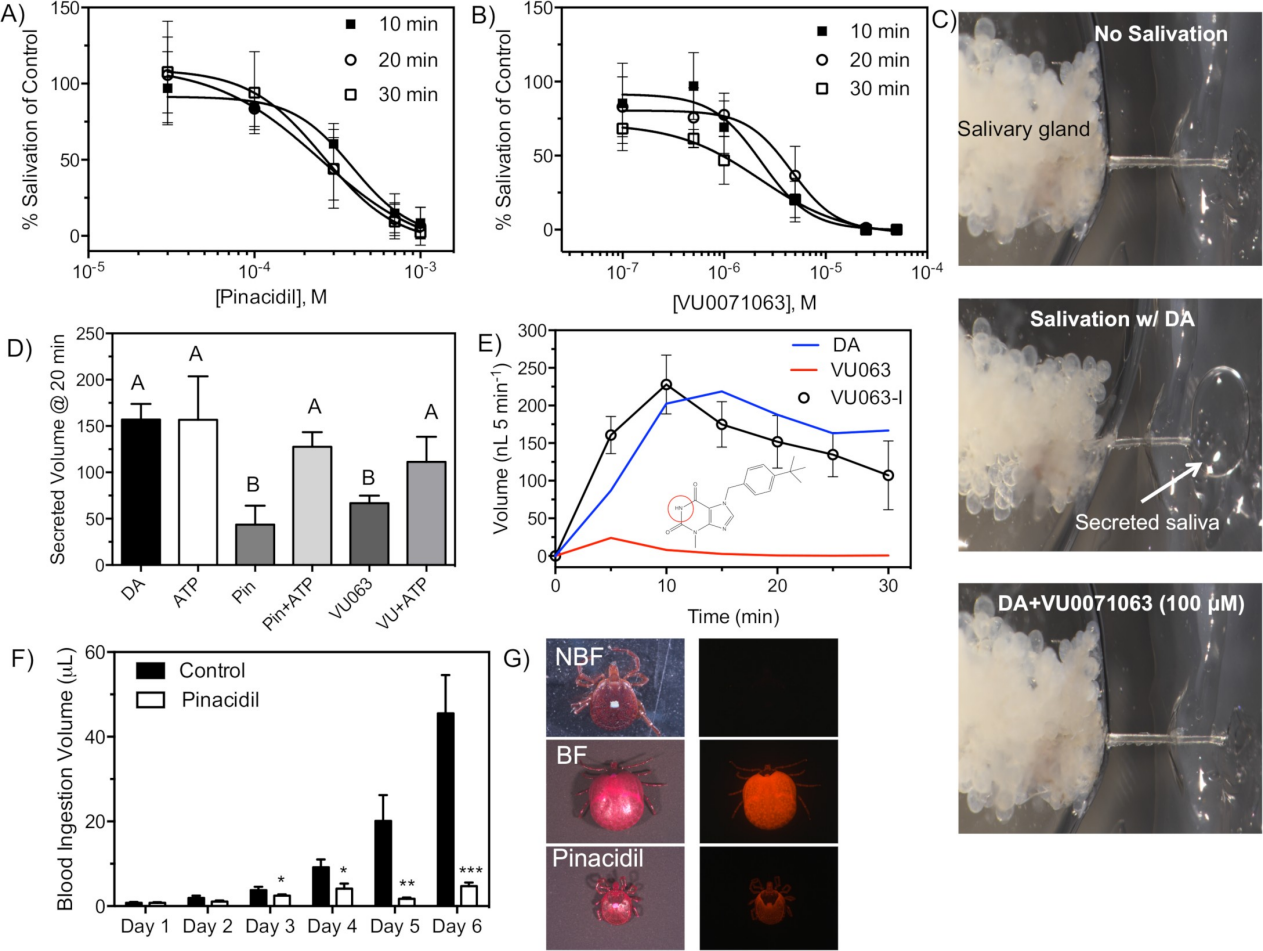
Influence of established K_ATP_ modulators to salivary gland function and blood feeding of *A*. *americanum*. Concentration dependent inhibition of the secretory activity from isolated *A*. *americanum* salivary glands with pinacidil **(A)** and VU0071063 **(B)** at 10 min (closed square), 20 min (open circle), and 30 min (open square) after dopamine-mediated stimulation of glands. For panels A-B, each data point represents a mean (n = 3–5) percent salivation of control and error bars represent SD, where each treated gland was compared to dopamine only treated gland excised from the same tick. **(C)** Representative images describing the reduced secretory activity after VU0071063 exposure. **(D)** K_ATP_ validation of the reduced secretory activity was determined by challenging pinacidil- and VU0071063- inhibition with ATP. Bars represent mean (n: 3–5) salivation volume at 20 minutes with error bars representing SD. Bars not labeled by the same letter represent statistical significance at P<0.05 as determined by a one-way ANOVA with multiple comparisons analysis compared to DA treated (control) glands. **(E)** VU063-I, the inactive analog of VU0071063, was used as for additional validation and the time course of fluid secretion from isolated salivary glands is shown with open circles for VU063-I and is compared to DA only (blue line) and VU0071063 only (red line). The VU063-I molecular structure is shown as an inset in panel E with the red circle highlighting the absence of the methyl group found on VU0071063. **(F)** Time course of the average blood ingestion between control and pinacidil exposed ticks. Bars represent mean ingestion volume and error bars represent SEM. Asterisks represent statistical significance with * P<0.05, ** P<0.01, and *** P<0.0001 as determined by an unpaired *t-*test. **(G)** Light (left column) and fluorescent (right column) images showing the ticks without ingestion (NBF), and ticks in blood only (BF), and pinacidil treated groups after six-day feeding.

In mammalian systems, the off-target effects of pinacidil and VU0071063 are minimal [54]. However, the possibility of modulating the activity of off-target proteins that may alter tick salivary gland activity remains present and therefore, we aimed to ensure the reduced secretory activity was indeed due to modulation of K_ATP_ channels. First, we generated concentration response curves to determine the concentration required to inhibit 50% (IC_50_) of the secretory activity to describe the potency of each molecule and ensure concentration dependency. Pinacidil was shown to be moderately potent at reducing the secretory activity of the *A*. *americanum* salivary gland with IC_50_ values in the mid micromolar range. At 10 minutes, pinacidil was shown to have an IC_50_ value of 389 μM (95% CI: 218–692 μM; Hillslope: -2.4; r^2^: 0.89) whereas the 20- and 30-minute IC_50_ values were found to be 1.56- and 1.50-fold reduced, respectively Fig 3A. The Kir activator VU0071063 was shown to be 169-fold more potent than pinacidil with an IC_50_ value of 2.3 μM (95% CI: 1.1–4.7 μM; Hillslope: -1.7; r^2^: 0.85) at 10 minutes. An increase in potency of VU0071063 was also observed at 20 and 30 minutes with IC_50_ values of 4.6 μM (95% CI 2.8–7.4 μM; Hillslope: -1.9; r^2^: 0.88) and 2.2 μM (95% CI: 1.1–4.7 μM; Hillslope: -1.0; r^2^: 0.9), respectfully (Fig 3B). Representative images of the secretory activity of the isolated salivary gland before stimulation, during stimulation, and after K_ATP_ channel exposure is shown in Fig 3C.

K_ATP_ channels are inhibited by the presence of ATP, which provides an avenue to ensure that the pharmacological activators are modulating K_ATP_ channels to induce a physiological response. In theory, the presence of ATP should irreversibly close the K_ATP_ channel and prevent pharmacological activation of the channel, thus reducing the potency of pinacidil and/or VU0071063 against the tick salivary gland. To test this hypothesis, the salivary gland was co-treated with ATP and pinacidil or VU0071063 to measure the changes in secretory activity. Exposure to 500 μM ATP did not influence the secretory activity of the isolated gland (Fig 3D) when compared to dopamine controls, whereas higher concentrations were shown to reduce the secretory activity. Importantly, the mean (n = 3–5) volume of secreted saliva after the isolated salivary gland was treated with 500 μM ATP and 300 μM pinacidil was found to be 127 ±9 nL / 5min, which was not statistically different from control secretion volumes (Fig 3D). Similarly, the volume of the secreted saliva after the isolated salivary gland was treated with 500 μM ATP and 1 μM VU0071063 was found to be 111 ± 17 nL / 5min, which was significantly increased from the volume secreted after 1 μM VU0071063 exposure, but not statistically different from control secretion volumes (Fig 3D). These data combined with the concentration dependency provide significant support that pinacidil and VU0071063 are indeed modulating the secretory activity through activation of K_ATP_ channels.

During the development of VU0071063, it was discovered that removal of the methyl group from the theophylline moiety dramatically decreased potency at the human Kir6.2/SUR1 channel (molecular structure shown in insert of Fig 3E). The significant loss of potency of the VU0071063 analog, termed VU063-I, provided an additional mechanism to ensure the reduction of salivary gland secretory activity after VU0071063 exposure was due to K_ATP_ channel activation. Indeed, exposure of the isolated tick salivary gland to VU063-I did not alter the secretory activity of tick salivary glands at any time point when compared the secretory activity of paired control glands (Fig 3E).

### Pinacidil and VU0071063 reduce blood ingestion and alter feeding behavior

Next, we aimed to translate the *in vitro* data to a biological response in the live tick and tested the hypothesis that inducing salivary gland failure will prevent tick feeding and blood ingestion. With a membrane feeding system optimized for *A*. *americanum* [51], control and pinacidil treated ticks were shown to imbibe nearly the same volume of blood at days 1 and 2, with mean (n = 3–9) ingestion of control and treated ticks 0.7 ± 0.2 μL and 1.3 ± 0.4 μL, respectively (Fig 1F). At 3, 4, 5, and 6 days of feeding, control ticks were shown to imbibe 1.5-, 2.2-, 12.1-, and 10.8-fold more blood when compared to ticks feeding on pinacidil-treated blood, respectively (Fig 3F). The total volume of blood ingestion by ticks exposed to pinacidil was significantly reduced (P<0.05) for each time point. Fluorescent images showing the reduced ingestion of blood are shown in (Fig 3G). We expected that VU0071063 would reduce the ability of actively feeding ticks to ingest blood with a greater efficacy than pinacidil, since VU0071063 was found to be significantly more potent at reducing the fluid secretion from the isolated salivary gland. Unfortunately, the increased mortality rate of VU0071063 (300 μM) exposed ticks (ca. 90% at day 2) prevented the ability to accurately determine the ingestion volume beyond day 1.

**Fig 4:**
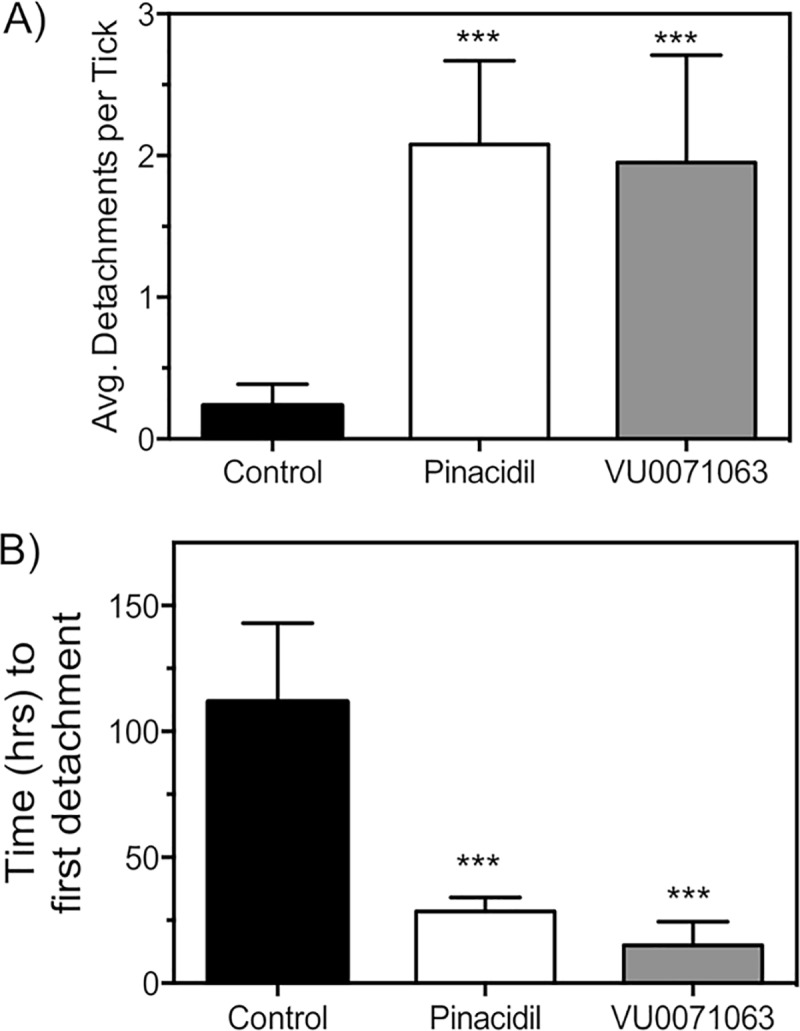
Influence of pinacidil and VU0071063 on blood feeding behavior of *A*. *americanum*. **(A)** Changes in the number of detachments per tick during a feeding cycle after exposure to K_ATP_ activators. **(B)** Time from the initial attachment to the membrane to the first detachment for control-, pinacidil, and VU0071063 treatment groups. Bars represent mean (n>30) and error bars represent SEM. Asterisks (***) represent statistical significance at P<0.001.

In addition to reduced blood ingestion, we analyzed the altered feeding behavior of ticks since horizontal transmission of bacterial pathogens does not occur until at least 12 hours of feeding has occurred [55], suggesting that an interruption of blood feeding (e.g. detachment) prior to this time point could occlude pathogen transmission. Individual ticks in the control group were found to detach from the membrane an average of 0.23 ± 0.1 times during the course of a complete feeding event (Fig 4A). Ticks feeding on pinacidil treated blood increased the number of detachment events per tick to an average of 2.1 ± 0.4 times per blood feeding event (Fig 4A), a statistically significant (P < 0.001) increase when compared to control groups. Similarly, ticks exposed to VU0071063 during blood feeding detached at a significantly (P<0.001) greater rate with an average of 1.9 ± 0.2 detachments (Fig 4A).

In addition to the total number of detachments, we analyzed the average time from initial attachment onto the membrane to the first detachment because the time of detachment has direct impact for pathogen transmission. A total of 5% of the control ticks were shown to detach during feeding and the first detachment was found to be 112 ± 18 hours (Fig 4B). Importantly, the time until first detachment was approximately 60–70% of the feeding time required to obtain a complete blood meal (168–200 hours or 7–9 days) with the artificial host membrane feeding system. On the contrary, ticks exposed to pinacidil or VU0071063 were found to feed for an average of 28 ± 3 hours and 15 ± 1.5 hours prior to the first detachment, a statistically significant (P<0.001) reduction when compared to control (Fig 4B).

### K_ATP_ channels regulate the osmoregulatory capacity of *Amblyomma americanum* salivary glands

**Fig 5:**
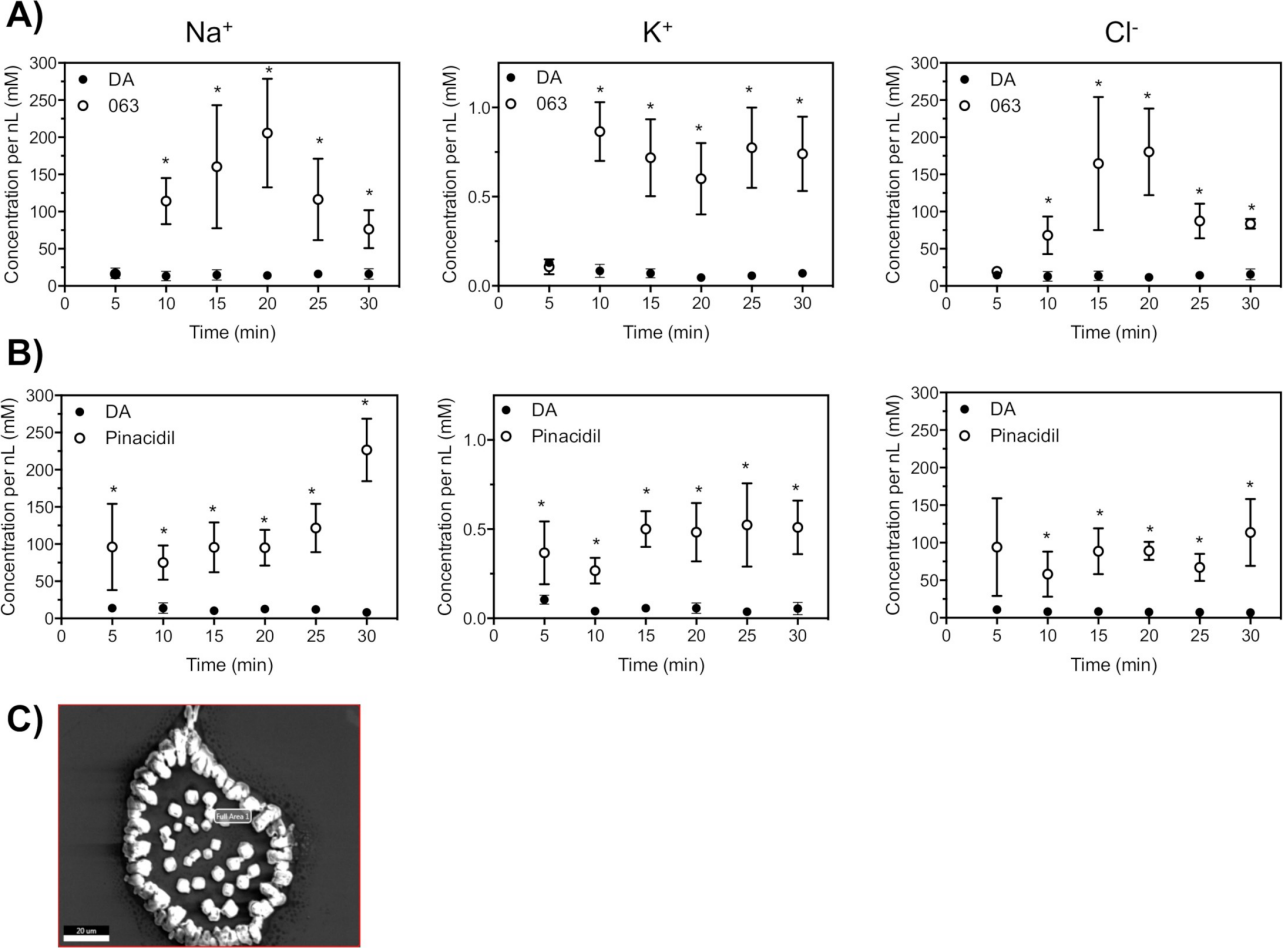
Elemental composition and relative concentration of secreted saliva from isolated *A*. *americanum* salivary glands. Qualitative determination of the concentration of Na^+^, K^+^, and Cl^-^ after VU0071063 **(A)** and pinacidil **(B)** treated salivary glands (open circles) compared to dopamine (DA; 100 μM) treated glands (closed circles). SEM/EDS was used to analyze each ion concentration from each time point. Asterisks represent statistical significance at P<0.05 as determined by an ANOVA-Tukey-Kramer HSD test. **(C)** Representative SED-EDS image of salt crystals from desiccated saliva droplet after treatment with 300 μM VU0071063. The scale bar represents 20 μM.

Elemental analysis of the secreted saliva from isolated salivary glands exposed to pinacidil (500 μM) and VU0071063 (5 μM) significantly altered the concentrations of Na^+^, K^+^, and Cl^-^ ions, indicating altered osmoregulatory capacity. The relative concentration of Na^+^, K^+^, and Cl^-^ was statistically (P<0.01) increased after exposure to VU0071063 for all time points except 5-minutes (Fig 5A), whereas all time points studied after pinacidil treatment was stastistically significant when compared to control ticks (Fig 5B). A representative crystalized salivary droplet is shown in Fig 5C. These data indicate that K_ATP_ channels represent a critical K^+^ ion conductance pathway in the *A*. *americanum* salivary gland and modulation of this pathway prevents fluid secretion (Figs 2, 3A and 3B), tick blood feeding (Fig 3F and 3G), and altered osmoregulatory capabilities (Fig 5).

### Exposure to K_ATP_ channel activators during blood feeding leads to tick mortality

**Fig 6:**
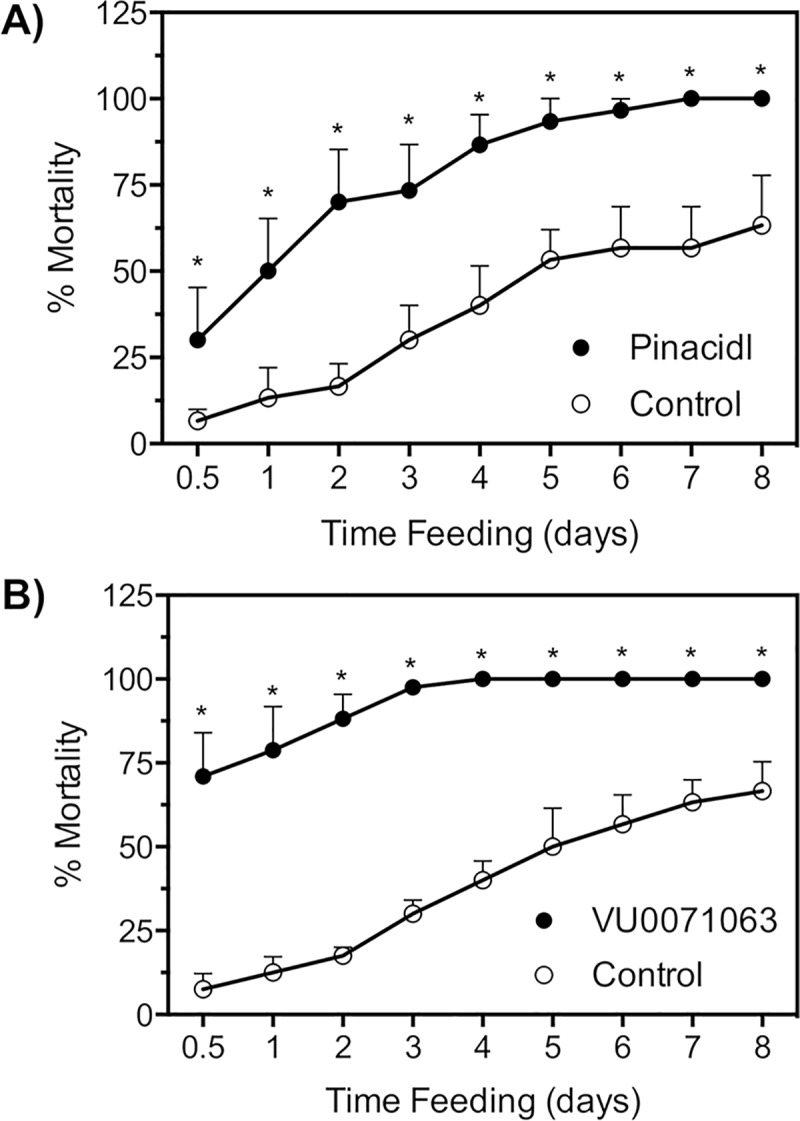
Influence of pinacidil and VU0071063 to mortality of *A*. *americanum*. Time course of mortality after exposure to pinacidil **(A)** and VU0071063 **(B)** compared to ticks exposed to vehicle control. Data points represent mean (n>30) mortality and error bars represent SEM. Asterisks (*) represent statistical significance at P<0.05 as determined by an unpaired *t*-test performed between the treatment and control group for each time point.

The time to effectively kill 50% of ticks (ET_50_) was found to be significantly different between pinacidil and control treatments with pinacidil ET_50_ being 1 day whereas the ET_50_ for control groups was 5 days. At day one, we observed a mean 13 ± 1.0% mortality in control groups and a 50 ± 8% mortality at day 1 of pinacidil fed ticks (Fig 6A). Further, mortality exceeded 90% at day 5 in pinacidil treatment groups whereas the same percent mortality was not reached in control groups until 13 days of feeding.

Similarly, ticks feeding on VU0071063 (300 μM) treated blood meals yielded mortality that was significantly greater than the mortality observed in control groups and at a faster rate when compared to pinacidil even though it was at a 3-fold lower concentration than pinacidil (Fig 6A and 6B). Statistical significance (P<0.05) was observed for mortality at each time point. The ET_50_ for VU0071063 was found to be less than 12 hours with 71 ± 4% mortality at the 12-hour time point. The ET_50_ for the control group was found to be 4.75 days, which is more than a 9.5-fold increase when compared to the ET_50_ of VU0071063 treated animals. Mortality for VU0071063 treated groups reached 100% 3-days after the start of feeding, whereas control ticks did not reach 75% mortality until day 8 of feeding (Fig 6B).

Ticks that fed on pinacidil- or VU0071063- treated blood were lethargic and had uncoordinated movements when they attempted to walk or move their chelicerae. Importantly, injection of pinacidil or VU0071063 into partially fed or non-blood fed adult ticks did not induce mortality or any signs of intoxication.

## Discussion

Tick saliva is critical for feeding of arthropods by carrying proteins that have immunomodulatory, anti-hemostatic, and anesthetic properties [17,22,56,57]. In addition to enabling feeding, arthropod saliva is the media through which pathogens are horizontally transferred to the vertebrate host. Further, the volume of secreted saliva is directly correlated to the severity of infection since most pathogens have an infectious dose and since saliva can affect immune cells to exacerbate infection [58]. This suggests that eliminating or reducing the volume of saliva secreted into the host will prevent feeding and reduce pathogen infectivity. Therefore, the objective of this study was to define the relevance of a conserved K^+^ ion channel to successful feeding of ticks to provide a platform for future research aimed at developing novel therapeutics to reduce or eliminate the health and economic burden from blood feeding of ticks and other arthropods.

Previous work has shown that inward potassium conductance is critical for mammalian salivary gland function [28,30,31] and may represent a target for inducing arthropod salivary gland failure [35,45]. The data presented in this study support the hypothesis that K_ATP_ channels in the *A*. *americanum* salivary gland are critical for feeding as we provide clear evidence that pharmacological activators of these channels reduce the secretory capacity of the salivary gland and hinder blood feeding. These data suggest that K_ATP_ channels regulate the epithelial physiology of the arthropod salivary gland function in a similar manner as Kir channels in mammalian salivary glands [30,31] and, from an applied perspective, represent a putative target site for the development of therapeutic agents that induce salivary gland failure to prevent feeding and/or osmoregulation to result in mortality.

In mammals, Kir channels represent a critical conductance pathway in multiple tissues, yet these channels have only recently gained traction as a physiologically and toxicologically relevant ion channel in insects [24,25,52,59]. Recent work on insect Kir channels suggests that these channels drive Malpighian tubule function by providing a transport pathway for K^+^ ions from the hemolymph to the Malpighian tubule lumen [36]. Importantly, *in vitro* and *in vivo* results indicate that small-molecule inhibitors specific for insect Kir channels disrupt K^+^ and fluid secretion at the level of the Malpighian tubules, leading to disruptions of hemolymph K^+^ and fluid homeostasis of the whole mosquito to result in mortality [24,25,36]. Mortality was attributed to the altered ion and fluid homeostasis and due to this, it has been proposed that Kir channels expressed in the Malpighian tubules represent a novel mechanism target site for arthropod control. Interestingly, the Malpighian tubules and salivary glands are physiologically related tissues as both tissues are a polarized epithelial tissue [60,61] that rely on potassium ion transport across membranes [62–64] to generate isosmotic fluid to form urine or saliva. Furthermore, the Kir-encoding gene of the mosquito Malpighian tubule is analogous to the Kir encoding gene in the salivary gland of *Drosophila* [65], which raised the intriguing possibility that Kir channels control the membrane potential and secretory activity of the arthropod salivary gland as it does in the mosquito Malpighian tubules [36]. Indeed, salivary gland specific knockdown of Kir1 mRNA or pharmacological inhibition of Kir1 channels resulted in a significant reduction of sucrose ingestion during *D*. *melanogaster* feeding [35]. Further, recent work has shown that inhibition of Kir1 channels in the brown planthopper (*Nilaparvata lugens*) reduces salivary and honeydew secretions [45]. These studies provide proof-of-concept that Kir channels represent an important channel for feeding and proper function of the arthropod salivary gland and represents a pharmacologically tractable target site to induce salivary gland and feeding failure in arthropod vectors.

Two classical Kir channel inhibitors, VU041 and VU625, did not influence the secretory activity of the tick salivary gland, which was surprising since these two molecules are highly potent inhibitors of mosquito Kir1 channels [24,25] and because Kir2.1, which is a constitutively active channel similar to insect Kir1, was identified to be partly responsible for spontaneous fluid secretion in mammalian salivary glands [31]. Although nucleotide or protein differences between mosquito and tick Kir channels may account for reduced efficacy of these molecules, we speculate the lack of activity of classical Kir channel modulators to tick salivation is due to different physiological requirements of blood feeding arthropods when compared to ruminant mammals or fruit flies, such as the requirement for ATP to stimulate blood feeding [66].

Since ATP is required for blood feeding in hematophagous arthropods, we hypothesized that blood feeding arthropods evolved the use of K_ATP_ channels instead of classical Kir channels to maintain the membrane potential and regulate the secretory activity of the salivary glands. Indeed, two structurally and mechanistically different activators of K_ATP_ channels were shown to reduce the secretory activity of the *A*. *americanum* salivary gland in a concentration-dependent manner. Importantly, opposite patterns of salivation were observed with activators and inhibitors at 5 minutes, the inhibition of salivation was concentration dependent, and the reduction of salivation in ticks after exposure to pinacidil/VU0071063 was negated with the application of ATP. These data provide substantial evidence that the reduced salivation is indeed due to modulation of K_ATP_ channels and supports the notion that K_ATP_ channels are required for salivary gland function and saliva secretion of *A*. *americanum*. However, it is important to note that although there is a close correlation between reduced feeding and reduced saliva secretion (Fig 3), it is conceivable that the reduced blood ingestion could be due to altered Kir/K_ATP_ channel function at the phagostimulant sensilla, the feeding pump muscles, the salivary gland, or any combination of these systems. Future work is needed to determine the role of K^+^ channels in each organ system as well as to functionally couple Kir channels, salivary gland function, and blood feeding.

In addition to salivary secretions, the tick salivary gland is responsible for maintaining a proper salt and water balance during blood feeding by returning about 65–70% of the fluid and ion content of the blood meal back into the host to alleviate the burden of the increased salts derived from the blood meal [23]. Kir channels have been shown to be major routes of K^+^ ion uptake in the mosquito Malpighian tubules, which is the osmoregulatory organ of mosquitoes, and pharmacological inhibition of these channels induced tubule failure and altered osmoregulatory capabilities, which lead to mosquito mortality [24,25,36]. Accordingly, we hypothesized that pharmacological activators of Kir channels in the tick salivary gland would alter the ion secretion rates and elemental composition of tick saliva. Indeed, elemental analysis of the secreted saliva with SEM-EDS from isolated salivary glands exposed to pinacidil (500 μM) and VU0071063 (5 μM) significantly altered the concentrations of Na^+^, K^+^, and Cl^-^ ions, indicating altered osmoregulatory capacity. The altered osmoregulatory capacity is important because strict regulation of the ion concentration in the hemolymph and hemoceal is essential for proper function of muscles, nerves, and other physiological systems. The dramatic increase in salt excretion during tick feeding led us to hypothesize that the altered osmoregulatory capacity of ticks when exposed to K_ATP_ agonists during blood feeding will lead to mortality in a similar manner to what has been described in mosquitoes [24,25]. Indeed, mortality was significantly (P<0.05) increased for ticks that fed on pinacidil treated blood meals when compared to vehicle control treatments.

The signs of intoxication prior to mortality were reminiscent of neural poisoning since the ticks that fed on chemically treated blood were lethargic and displayed uncoordinated movements. Yet, injection of pinacidil or VU0071063 into partially fed or unfed ticks did not induce toxicity or any signs of intoxication. This difference indicates the mortality from exposure to K_ATP_ modulators during blood feeding is likely resultant from reduced ion concentrations in the tick hemolymph stemming from altered osmoregulatory capacity through the salivary gland, similar to mosquito mortality described after Malpighian tubule failure from Kir channel inhibition [25,52]. Taken together, the osmoregulatory and toxicity data indicate the possibility of developing novel mechanism acaricides by targeting ion transport and ion conductance pathways in the tick salivary gland that are functionally linked to establishing or maintaining transepithelial ionic balance.

In addition to the essential role the tick salivary gland has to blood feeding and osmoregulation, the volume of saliva secreted into the host during feeding is directly correlated to pathogen transmission and disease severity [58,67]. Thus, the reduction in secretory activity of the salivary gland after exposure to Kir/K_ATP_ modulators led to the logical assumption that horizontal transmission of a pathogen will be negated or reduced when the tick is exposed to Kir/K_ATP_ modulators during feeding. The dynamics of pathogen transmission have been studied extensively in ticks; and *A*. *aureolatum* was shown to successfully transmit a virulent strain of *Rickettsia rickettsii* to a vertebrate host after a feeding period of approximately 12–16 hours [55], suggesting that inducing detachment or inducing mortality within 12–16 hours of feeding would likely prevent pathogen transmission for this vector and pathogen. Importantly, K_ATP_ agonists drastically reduced saliva secretions (Figs 2, 3A and 3B), reduced uninterrupted feeding time to approximately 15 hours (Fig 4), and dramatically reduced blood ingestion and presumably, the saliva secretions into host (Fig 3F) when exposed to pinacidil. Since pathogen acquisition by the arthropod vector is directly correlated to blood intake, it is plausible to suggest that ticks exposed to K_ATP_ modulators during feeding will acquire fewer infectious particles since the tick intakes less blood (Fig 3F). Similarly, reduced salivary output is correlated to reduced disease severity because saliva can increase pathogen infection within a vertebrate host through effects on immune cells [58] and since reduced salivation yields less transmission of infectious particles to the host [67]. Further, it would be of great benefit to induce mortality with the same intervention method that is used to interrupt the dynamics of pathogen transmission. Indeed, pinacidil and VU0071063 increased the rate of mortality with VU0071063 inducing 75% mortality at 12 hours of feeding, which is believed to be the minimum feeding time required for bacterial pathogen transmission [55]. Therefore, these data suggest that K_ATP_ channel activators are likely to reduce or prevent pathogen transmission from *A*. *Americanum* and likely other species of hard ticks. Additional studies analyzing the horizontal transmission and acquisition of bacterial pathogens are required to validate this hypothesis.

The data indicate that the pharmacological profile of tick K_ATP_ channels is different when compared to human K_ATP_ channels, since pinacidil and VU0071063 both prevented salivation, but are selective for different SUR proteins in humans [53]. In mammals, K_ATP_ channels consist of multiple heteromeric combinations of Kir6.x and SURs that result in different pharmacological sensitivities and reflect the various K_ATP_ channels in native tissues. For instance, glibenclamide blocks the Kir6.2/SUR1 channel and is significantly less potent against Kir6.2/SUR2A and Kir6.2/SUR2B [68], whereas tolbutamide inhibits Kir6.2/SUR1 currents with high affinity, but does not inhibit Kir6.2/SUR2A [68]. Similarly, VU0071063 and diazoxide selectively activate the Kir6.2/SUR1 channel, whereas pinacidil preferentially activates Kir6.1/SUR2 channels [54]. Yet, in ticks diazoxide did not influence salivation whereas pinacidil and VU0071063 reduced secretory activity. The differences in pharmacological profiles between arthropods and humans suggest a unique heteromeric assembly of Kir and SUR constructs that can be exploited for the development of therapeutic agents that are highly specific for the arthropod K_ATP_ channel over the K_ATP_ mammalian channel, enabling vaccination or treatment of hosts with chemical modulators.

Determining the specific functional role of Kir channels at the cellular level of the salivary gland is important for the subsequent development of products aiming to prevent salivation and feeding and should be a focus of future work. Based on the role of Kir channels in mosquito Malpighian tubules [36,38] and in mammalian salivary glands [29,31], we speculate Kir/K_ATP_ channels are responsible for maintenance of the acinar membrane physiology that is necessary for function of downstream neuroendocrine systems that regulate fluid secretion across epithelia, such as dopamine. The membrane potential is known to control calcium mobilization, which stimulates prostaglandin E_2_ and subsequent secretion of bioactive salivary proteins [69]. Therefore, we speculate that Kir/K_ATP_ channels indirectly regulate calcium mobilization by regulating the membrane potential of the acini. For instance, K_ATP_ channels are open during times of low metabolic activity (i.e. low ATP/ADP ratio), which results in hyperpolarization of the membrane that prevents flux of calcium ions through voltage-gated calcium channels [70]. Therefore, pharmacological activation of K_ATP_ channels would lead to hyperpolarization of the salivary gland acini membrane, preventing calcium release and inhibiting gland function, which is supported by our data (Figs 2 and 3). However, the precise role of Kir channels in salivary gland function and their involvement with maintaining intracellular currents is difficult to ascertain without electrophysiological data describing how these channels interface with other ion channels and neuroendocrine systems.

This translational study highlights the critical role K_ATP_ channels provide in tick salivary gland function and the data suggest these proteins, and likely other membrane bound proteins linked to salivary gland function, represent novel therapeutic targets for measures that can be applied to reduce tick populations and/or tick vectored pathogens. These data support the notion that inward K^+^ conductance pathways in the tick salivary gland represent a putative therapeutic target ripe for development. Further, tick Kir/K_ATP_ channels appear to have maintained their fundamental role in animal physiology but the different pharmacological profile of K_ATP_ modulators between arthropods and humans suggests different protein composition that highlights the likelihood to develop selective agents of control that have low affinity to Kir channels of human, beneficial (e.g. honey bee), or non-target species. The data presented in this study provide insights of broad interest given the importance of insect feeding to the worldwide health and economic burden resulting from arthropod feeding.
